# Cardiac Comorbidity Burden and Post-Liver Transplant Outcomes: A Propensity-Matched Multicenter Analysis

**DOI:** 10.3390/jcm15166260

**Published:** 2026-08-13

**Authors:** Noor Albusta, Sara Isa, Ali Bosta, Rehab Almarzooq

**Affiliations:** 1 Department of Internal Medicine, Lahey Hospital and Medical Center, Burlington, MA 01805, USA; 2 School of Medicine, Royal College of Surgeons in Ireland, Medical University of Bahrain, Adliya, Busaiteen P.O. Box 15503, Bahrainaalbosta@hospitals.gov.bh (A.B.); 3 Medical Department, Salmaniya Medical Complex, Manama P.O. Box 12, Bahrain; rmarzooq1@hospitals.gov.bh

**Keywords:** liver transplantation, coronary artery disease, heart failure, atrial fibrillation, MASH, MACE, propensity score matching, cardiac comorbidity, mortality, outcomes

## Abstract

**Background/Objectives:** Cardiac comorbidities are increasingly common among liver transplant candidates, particularly those with metabolic dysfunction-associated steatohepatitis (MASH)-related cirrhosis. Although the Liver Transplant Comorbidity Index identifies coronary artery disease (CAD) as a predictor of post-transplant mortality, the impact of overall cardiac comorbidity burden on early outcomes after liver transplantation remains unclear. We evaluated the association between pre-transplant cardiac comorbidities and early post-transplant outcomes, with emphasis on MASH-related cirrhosis. **Methods:** We performed a retrospective cohort study using the TriNetX US Collaborative Research Network. Adults undergoing first-time isolated liver transplantation through May 2026 were included. Pre-transplant CAD, heart failure (HF), and atrial fibrillation (AF) documented within 12 months before transplantation were identified using ICD-10-CM codes. Patients were categorized by cardiac comorbidity burden (0–3 conditions). Recipients with any cardiac comorbidity underwent 1:1 propensity score matching to those without cardiac disease using 16 baseline demographic, clinical, and laboratory variables, including MELD-Na. The estimand was the average treatment effect in the treated patients. Primary outcomes comprised 30- and 90-day all-cause mortality. Secondary outcomes included a prespecified restricted major adverse cardiac event (MACE) composite, limited to hard endpoints (death, myocardial infarction, cardiac arrest, ischemic stroke), and a broader composite, i.e., acute kidney injury, prolonged mechanical ventilation, vasopressor requirement, renal replacement therapy, ICU and hospital length of stay, and 90-day readmission. **Results:** Among 5124 recipients, 986 (19.2%) exhibited at least one cardiac comorbidity. After matching, 974 patients remained in each group. Pre-transplant cardiac comorbidity was associated with higher 30- and 90-day mortality and increased risks of all secondary outcomes. The association with MACE persisted but was attenuated when restricted to hard endpoints (90-day RR 1.55; 95% CI 1.19–2.03) when compared with the broad composite (RR 1.75; 95% CI 1.40–2.18). MASH recipients with cardiac comorbidities experienced numerically higher event rates than did non-MASH recipients, but interaction estimates were imprecise and non-significant. In separate matched analyses, AF was most strongly associated with MACE, whereas CAD showed the strongest association with mortality. **Conclusions:** Pre-transplant cardiac comorbidity burden is associated with worse early post-transplant outcomes. Although MASH-cirrhosis recipients experienced numerically higher event rates, exploratory subgroup analyses did not demonstrate statistically significant differences from the non-MASH recipients. These findings may help refine cardiac risk prediction and perioperative planning, but they do not establish that intensified cardiac risk stratification or perioperative optimization improve outcomes. Prospective studies incorporating detailed cardiac, donor, operative, frailty, and medication data are needed to validate these associations and determine whether targeted risk-stratification and perioperative strategies can improve post-transplant outcomes.

## 1. Introduction

Liver transplantation is the definitive treatment for selected patients with decompensated cirrhosis, acute liver failure, and certain primary hepatic malignancies. The underlying diseases leading to transplantation are heterogeneous and include alcohol-associated liver disease, metabolic dysfunction-associated steatohepatitis (MASH), chronic viral hepatitis, autoimmune and cholestatic liver diseases, and cryptogenic cirrhosis. These etiologies differ not only in their hepatic manifestations but also in their associated extrahepatic comorbidity profiles, which may substantially influence perioperative and post-transplant outcomes.

The clinical profile of liver transplant candidates has changed considerably during the past two decades. Improvements in antiviral therapy have reduced the relative contribution of chronic hepatitis C, whereas alcohol-associated liver disease and metabolic dysfunction-associated steatotic liver disease have become increasingly prominent indications for transplantation. In particular, patients with MASH frequently exhibit obesity, type 2 diabetes, hypertension, dyslipidemia, chronic kidney disease, and clinically apparent or subclinical cardiovascular disease. As a result, contemporary transplant programs are evaluating older and more medically complex candidates than was the case in previous eras.

Transplant candidacy has also evolved from reliance on isolated age limits or individual comorbidities toward a multidisciplinary assessment of physiological reserve, frailty, organ-specific risk, expected post-transplant survival, and the potential reversibility or treatability of comorbid conditions. Cardiovascular disease does not invariably preclude transplantation, but it remains a major determinant of perioperative risk and candidate selection. Therefore, defining how the type and cumulative burden of pre-transplant cardiac disease affects early outcomes is increasingly important for informed candidate selection, counseling, and perioperative planning.

Cardiovascular disease is a leading cause of morbidity and mortality following liver transplantation (LT), with major adverse cardiovascular events (MACE) occurring in approximately 8% and 11% of recipients within 30 and 90 days after transplantation, respectively [1,2]. The perioperative period imposes substantial cardiopulmonary stress through hypovolemia, vasoplegia, vascular clamping, and graft reperfusion, which may unmask subclinical cardiac dysfunction and precipitate adverse events in recipients with pre-existing cardiovascular disease [3,4]. As the LT population has shifted toward older recipients with a greater burden of metabolic comorbidities—driven in part by the increasing prevalence of metabolic dysfunction-associated steatohepatitis (MASH) as an indication for transplantation—the prevalence of pre-transplant cardiac disease has increased correspondingly [5,6].

Coronary artery disease (CAD) affects approximately 16% of LT candidates and has been associated with increased all-cause and cardiovascular-specific mortality following transplantation [7,8]. In a single-center study of 693 LT recipients, patients with pre-existing CAD experienced significantly higher all-cause mortality than did those without CAD (26.7% vs. 9.6%; *p* < 0.01). Cardiovascular events accounted for 52.5% of deaths among patients with CAD, and the coexistence of nonalcoholic steatohepatitis (NASH) and CAD was associated with the poorest early post-transplant survival [9]. Atrial fibrillation (AF), which has a pooled pre-transplant prevalence of approximately 3–5% and a post-operative incidence of 7–10%, has been associated with a 2.3-fold increase in mortality and a 5.2-fold increase in cardiovascular complications [10,11]. Heart failure (HF), whether pre-existing or newly developed, occurs in approximately 10% of LT recipients and is independently associated with mortality. Early-onset HF may reflect perioperative cardiovascular stress, whereas late-onset HF is more commonly associated with underlying coronary atherosclerosis [2].

The recently developed Liver Transplant Comorbidity Index (LTCI), derived from the multicenter Functional Assessment in Liver Transplantation (FrAILT) study, integrates frailty, CAD, hepatocellular carcinoma, renal dysfunction, and diabetes into a composite score that predicts 3-year post-transplant mortality [12]. Notably, CAD emerged as a major component of the index, with moderate- and high-risk LTCI groups demonstrating 3-year mortality rates of 13% and 20%, respectively, compared with 7% among low-risk recipients [12]. However, the LTCI does not include HF or AF as independent variables, and the individual and cumulative contributions of specific cardiac comorbidities to early post-transplant outcomes—including MACE, acute kidney injury (AKI), prolonged mechanical ventilation, and hemodynamic instability—have not been systematically evaluated in a large multicenter cohort.

Several knowledge gaps persist. First, while individual cardiac comorbidities have been studied in isolation, the dose–response relationship between cumulative cardiac comorbidity burden (0, 1, 2, or 3 comorbidities) and early post-transplant outcomes has not been evaluated. Second, whether the association between cardiac comorbidities and adverse outcomes differs between MASH-cirrhosis and non-MASH etiologies remains incompletely characterized, despite the known higher cardiovascular risk profile of MASH patients [5,6]. Third, the relative contributions of CAD, HF, and AF to specific early outcomes—mortality, MACE, AKI, and respiratory complications—have not been directly compared within the same cohort. Fourth, the AASLD/AST Practice Guideline for adult liver transplantation candidate evaluation emphasizes the importance of pre-transplant cardiac evaluation but acknowledges the absence of a universally accepted screening algorithm, highlighting the need for data to inform risk stratification [12]. 

To address these gaps, we conducted a large multicenter retrospective cohort study using the TriNetX US Collaborative Research Network. The objectives of this study were to evaluate the association between pre-transplant cardiac comorbidity burden and a broad spectrum of early post-transplant outcomes, compare outcomes between MASH and non-MASH etiologies, and assess the relative contributions of individual cardiac comorbidities to specific clinical outcomes following LT.

## 2. Materials and Methods

### 2.1. The Data Source and Study Design

We performed a retrospective cohort study using the TriNetX US Collaborative Research Network, a federated real-world electronic health record database and analytics platform provided by TriNetX, LLC (Cambridge, MA, USA). The final query was executed on 31 May 2026. Eligible first-time isolated liver transplantations were identified through 28 February 2026, allowing at least 90 days of potential follow-up before the data cutoff. At the time of the query, the network included de-identified data from participating US academic medical centers, integrated healthcare systems, and community healthcare organizations; the identities of individual contributing organizations were not available to the investigators.

Available data include demographics, diagnoses coded using ICD-10-CM, procedures coded using CPT and ICD-10-PCS, medications, laboratory values, vital signs, and healthcare encounters. The index date was defined as the date of the first qualifying isolated liver transplantation. Cardiac comorbidities and baseline diagnoses were assessed during the 12 months before transplantation; laboratory values during the 90 days before transplantation; and outcomes during prespecified 7-, 30-, and 90-day postoperative windows. Mortality was identified from death information recorded within participating healthcare organizations. Because the completeness of external mortality linkage was not available, deaths occurring outside the network may not have been captured.

Multiple qualifying transplant procedure codes recorded for the same patient on the same calendar date were treated as a single transplant episode, and the earliest qualifying transplant date was used as the index event. Patients with evidence of liver transplantation before the index date were excluded as retransplant recipients. Eligibility and exclusion criteria were applied sequentially, resulting in 5124 eligible recipients from an initial cohort of 6482 patients with liver-transplant procedure codes.

Because TriNetX is a federated EHR network, patient-level chart review, detailed donor information, intraoperative variables, echocardiographic data, ventilator flow-sheet data, urine output, and center-specific transplant practices were not uniformly available. Therefore, exposures, covariates, and outcomes were defined using structured diagnosis, procedure, medication, laboratory, and encounter data. All ICD-10-CM, ICD-10-PCS, and CPT codes used to define exposures, covariates, etiologies, and outcomes are stated in full in Section 2.2, Section 2.3 and Section 2.4; no additional codes were used.

This study did not require institutional review board approval because the data were de-identified and compliant with the Health Insurance Portability and Accountability Act. The study was conducted in accordance with the Strengthening the Reporting of Observational Studies in Epidemiology guidelines for observational research.

### 2.2. Study Population and Cohort Definitions

We identified all adult patients (≥18 years) who underwent first-time, isolated liver transplantation using ICD-10-PCS codes (0FY00Z0, 0FY00Z1, 0FY00Z2) and CPT code 47135. The index date was defined as the date of the transplant procedure.

Patients were classified into two primary cohorts based on the presence or absence of documented pre-transplant cardiac comorbidities within 12 months before the index transplant date.

The cardiac comorbidity cohort comprised patients with at least one of the following ICD-10-CM codes documented within 12 months before transplantation: coronary artery disease (I25.10, I25.11x, I25.2, I25.5, I25.6, I25.7x, I25.81x, I25.89, I25.9), heart failure (I50.1, I50.20–I50.23, I50.30–I50.33, I50.40–I50.43, I50.8x, I50.9), or atrial fibrillation/flutter (I48.0, I48.1, I48.2, I48.91). Patients were further subclassified by the number of cardiac comorbidity domains present (1, 2, or 3) for dose–response analysis.

The no cardiac comorbidity cohort comprised patients without any of the above codes documented within 12 months before transplantation.

Exclusion criteria, applied uniformly to both cohorts, were are follows: age < 18 years; incomplete demographic data, defined as missing age, sex, or race; multiorgan transplantation, including simultaneous liver–kidney, liver–heart, or liver–lung transplantation; retransplantation, identified by prior liver transplantation procedure codes or documentation of repeat liver transplantation before the index date when available in the structured EHR record; acute/subacute hepatic failure due to trauma, acetaminophen overdose, or other acute toxic etiologies (ICD-10-CM K71.1x, T39.1x); and insufficient baseline data for propensity matching, defined as missing ≥3 of the prespecified matching variables.

### 2.3. Baseline Characteristics and Covariates

Sixteen baseline covariates were collected for propensity score matching and multivariable adjustment, including demographic variables (age, sex, and race/ethnicity [White, Black, Hispanic]); comorbidities documented within 12 months before transplant (type 2 diabetes mellitus [E11.x], hypertension [I10–I16], chronic kidney disease [N18.x], hepatic encephalopathy [K72.x, G93.4x], ascites [R18.x], hepatocellular carcinoma [C22.0], obesity [E66.x], hyperlipidemia [E78.x], and pre-transplant dialysis [Z99.2]); albumin; and MELD-Na.

The MELD-Na score was calculated from bilirubin, INR, creatinine, and sodium levels using the standard OPTN formula. Bilirubin, INR, creatinine, and sodium levels were not entered separately alongside MELD-Na in the propensity score model because they are its algebraic components and would introduce redundant, collinear predictors. These laboratory variables are reported in Table 1 solely as post-match balance diagnostics.

Baseline laboratory values were defined as the measurement closest to and preceding the index date within the 90-day baseline window; worst and averaged values were not used. No imputation was performed. Patients missing fewer than three matching variables were retained, with missingness handled as a separate indicator category within the propensity model; patients missing three or more matching variables were excluded (*n* = 143, 2.7% of otherwise eligible recipients).

Liver disease etiology was assigned using a prespecified hierarchy applied in the following order. First, any code for viral hepatitis (B18.x, K73.x) or alcohol-associated liver disease (K70.x) took precedence and excluded the classification as MASH, irrespective of metabolic risk factor codes. Second, among remaining patients, those with K75.81 plus a cirrhosis code (K74.x) were classified as definite MASH-cirrhosis. Third, those meeting only the metabolic-risk-factor-plus-cirrhosis criterion (obesity, type 2 diabetes, or dyslipidemia with K74.x, without K75.81) were classified as probable MASH. Remaining patients were classified as autoimmune/cholestatic (K75.4, K74.3, K83.01) or other/cryptogenic. Of 436 patients classified as MASH, 187 (42.9%) met the definite definition and 249 (57.1%) the probable definition. A sensitivity analysis restricted to the definite (strict) MASH definition is reported in Section 3.10.

### 2.4. Study Endpoints

Primary outcome: All-cause mortality at 30 and 90 days after liver transplantation.

Secondary outcomes:

- Major adverse cardiac events (MACE). MACE was defined a priori in two ways. The primary, restricted definition (“hard MACE”) comprised only endpoints that cannot overlap with the exposure definitions, i.e., all-cause death, myocardial infarction (I21.x), cardiac arrest (I46.x), and ischemic stroke (I63.x). A secondary, broad definition additionally included postoperative HF or arrhythmia, counted only when (i) the patient had no code for that condition during the 12-month pre-transplant baseline window (incident event), or (ii) the event coincided with an inpatient encounter carrying a first-listed cardiac diagnosis or a cardiac-specific intervention such as cardioversion, antiarrhythmic infusion, or mechanical circulatory support (clinically active event). Recipients with baseline HF or AF contributed to the broad composite only through this rule. Individual components of both composites are reported separately in Table 2B.
- Acute kidney injury (AKI). i.e., ICD-10-CM N17.x within the specified postoperative window. KDIGO-based staging was not used because granular urine output data and sufficiently time-stamped serial creatinine values are not uniformly available across participating organizations. Because coded AKI is expected to capture predominantly clinically recognized moderate-to-severe events, peak inpatient creatinine and change in creatinine from baseline to peak were prespecified as co-secondary continuous renal endpoints.
- Prolonged mechanical ventilation, i.e., ICD-10-PCS 5A1955Z during the index hospitalization, identifying ventilation lasting more than 96 consecutive hours. CPT 94002 and 94003 were not used because they represent daily ventilator-management services.
- Renal replacement therapy, i.e., ICD-10-PCS 5A1D70Z/5A1D80Z or CPT 90935/90937/90945/90947.
- ICU length of stay; hospital length of stay; 90-day all-cause readmission.

### 2.5. Statistical Analysis

Baseline characteristics were compared using Chi-square tests for categorical variables and Student’s *t*-tests or Wilcoxon rank-sum tests for continuous variables.

The estimand was the average treatment effect in the treated (ATT), i.e., the effect of pre-transplant cardiac comorbidity among recipients who had such comorbidity. Propensity scores were estimated by logistic regression, with cardiac comorbidity as the dependent variable and the 16 prespecified covariates entered as main effects only, without interaction or higher-order terms. Matching was achieved using the 1:1 nearest-neighbor method, without replacement, with a caliper of 0.1 standard deviations of the logit of the propensity score. Balance was assessed using standardized mean differences (SMD), with values < 0.10 considered adequate. Pre- and post-match SMDs for every covariate are reported for the primary match and for each individual-comorbidity match; propensity-score overlap is summarized numerically in Section 3.1.

For each binary outcome, relative risks (RRs) and risk differences (RDs) with 95% confidence intervals (CIs) were calculated at predefined intervals. All outcome analyses accounted for the matched design: binary outcomes were analyzed using matched-pair methods, and Cox models used robust (sandwich) standard errors clustered on a matched pair.

Time zero for all time-to-event analyses was the index transplant date. Follow-up extended from time zero to the first of outcome occurrence, death, last recorded encounter within the network, or the end of the relevant 30- or 90-day window. Patients with no recorded post-index encounter were censored at day 0 and are reported separately (*n* = 18 cardiac, *n* = 21 no cardiac). For nonfatal outcomes, death was treated as a competing event, and Fine–Gray subdistribution hazard models are reported alongside cause-specific Cox estimates. Cox models were fitted within the TriNetX analytics platform survival module, which implements Cox proportional hazards regression on the matched cohort; the proportional hazards assumption was assessed using scaled Schoenfeld residuals with global and covariate-specific tests. Fixed 7-, 30-, and 90-day risks are reported as matched risk differences and relative risks rather than derived from the survival models because these are fixed-horizon risks in a cohort with near-complete short-term follow-up.

Distributions of hospital length of stay, ICU length of stay, and ventilator duration were right-skewed on inspection; these are summarized as medians with interquartile ranges and compared using quantile regression accounting for matched pairs, with means retained for comparability with prior literature.

No formal multiplicity adjustment was applied to the primary analysis. The 30- and 90-day mortality comparisons in the primary matched cohort were prespecified as primary endpoints; all other outcomes, time points, individual-comorbidity analyses, dose–response tests, and MASH/non-MASH analyses are secondary or exploratory and are interpreted as hypothesis-generating. The Benjamini–Hochberg false-discovery-rate (FDR) adjustment was applied across the secondary outcome family, and adjusted *p* values are reported in Section 3.3.

Sensitivity and subgroup analyses included:

1. MASH vs. non-MASH subgroup analysis, exploratory. Interaction was assessed on the multiplicative scale, with ratios of relative risks and 95% CIs reported alongside interaction *p* values.
2. Dose–response analysis across comorbidity burden categories (0, 1, 2, 3) using the Cochran–Armitage test for trend.
3. Individual cardiac comorbidity analysis, i.e., separate propensity-matched analyses for CAD, HF, and AF vs. no cardiac comorbidity. Because patients could contribute to more than one comparison, these estimates are not mutually adjusted.
4. MELD 3.0 sensitivity analysis.

Statistical significance was defined as a two-sided *p*-value < 0.05. All analyses were conducted within the TriNetX analytics platform.

## 3. Results

### 3.1. Study Population

**Figure 1:**
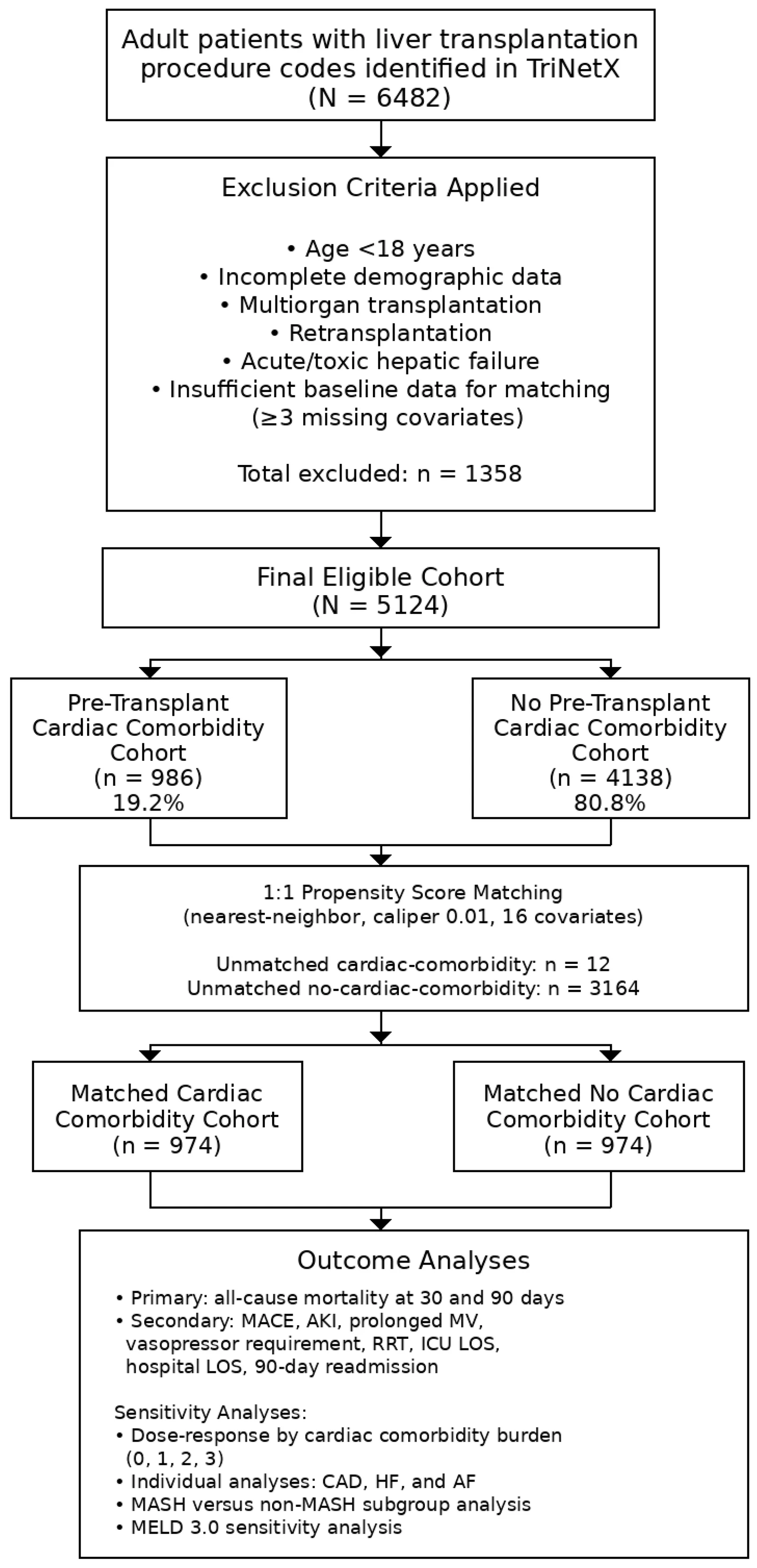
Patient selection and propensity score matching flow diagram for adult liver transplant recipients with and without pre-transplant cardiac comorbidities in the TriNetX US Collaborative Research Network. Abbreviations: AF = atrial fibrillation; AKI = acute kidney injury; CAD = coronary artery disease; HF = heart failure; ICU = intensive care unit; LOS = length of stay; MACE = major adverse cardiac events; MASH = metabolic dysfunction-associated steatohepatitis; MELD = Model for End-Stage Liver Disease; MV = mechanical ventilation; RRT = renal replacement therapy.

A total of 6482 adult patients with liver transplantation procedure codes were identified in the TriNetX US Collaborative Research Network. After sequential application of exclusion criteria, 5124 patients comprised the final eligible cohort: 986 (19.2%) with at least one pre-transplant cardiac comorbidity and 4138 (80.8%) without. Among those with cardiac comorbidities, 648 (65.7%) had one, 264 (26.8%) had two, and 74 (7.5%) had three. The most prevalent individual cardiac comorbidity was CAD (*n* = 587, 59.5%), followed by AF (*n* = 384, 38.9%) and HF (*n* = 341, 34.6%). Before propensity score matching, recipients with cardiac comorbidities were older and had higher prevalences of type 2 diabetes, hypertension, CKD, obesity, hyperlipidemia, pre-transplant dialysis, higher MELD-Na scores, higher creatinine levels, and lower albumin levels compared with the results for recipients without cardiac comorbidities. After 1:1 PSM, 974 patients remained in each group, and all measured baseline covariates were well balanced, with all post-matching standardized mean differences < 0.10 (Table 1). The patient selection process is illustrated in Figure 1.

### 3.2. Baseline Characteristics

Before PSM, the cardiac comorbidity cohort was significantly older, more likely to be male, and had a higher prevalences of type 2 diabetes, hypertension, CKD, obesity, hyperlipidemia, and pre-transplant dialysis, as well as higher MELD-Na scores and worse laboratory parameters. After PSM, all 16 covariates were well balanced (all SMD < 0.10) (Table 1).

### 3.3. Primary and Secondary Outcomes

At 30 days, all-cause mortality was significantly higher in the cardiac comorbidity cohort (RR 1.82; 95% CI: 1.28–2.59; *p* = 0.003). This association persisted at 90 days (RR 1.61; 95% CI: 1.24–2.09; *p* < 0.002). Cardiac comorbidity was significantly associated with increased risk of MACE, AKI, prolonged mechanical ventilation, vasopressor requirement, and RRT at all assessed time points. Ninety-day readmission was also significantly higher in recipients with cardiac comorbidities (Table 2A).

Using the prespecified restricted hard-endpoint composite, which excludes any component overlapping with the exposure definitions, the association persisted but was attenuated relative to the broad composite: 30-day RR 1.65 (95% CI 1.18–2.30) and 90-day RR 1.55 (95% CI 1.19–2.03) vs. 1.97 and 1.75, respectively, for the broad composite. Component-level counts are shown in Table 2B.

After Benjamini–Hochberg FDR adjustment across the 14 secondary outcome comparisons shown in Table 2, all associations significant at the nominal level remained significant (adjusted *p* ≤ 0.008 for MACE, AKI, prolonged mechanical ventilation, vasopressor requirement, and RRT), with the exception of 90-day readmission, which was attenuated to borderline significance (nominal *p* = 0.002; adjusted *p* = 0.061).

### 3.4. Length of Stay and Continuous Outcomes

Cardiac comorbidity was associated with significantly longer hospital and ICU stays, higher peak creatinine, and longer ventilator duration among ventilated patients (Table 3). Because several of these variables were recorded only in subsets of the matched cohort, denominators are reported for each outcome, and the analyses are conditional on the variable being recorded.

### 3.5. Cox Proportional Hazards Model

Cox regression, using robust standard errors clustered on matched pair, confirmed higher hazards for all primary and secondary outcomes in recipients with cardiac comorbidities (Table 4). The proportional hazards assumption was satisfied in all models (global scaled Schoenfeld residual test, *p* = 0.14–0.82; no covariate-specific test, *p* < 0.05). Accounting for death as a competing event, Fine–Gray subdistribution hazard ratios for nonfatal outcomes were modestly attenuated but directionally unchanged: AKI 1.31 (95% CI 1.16–1.48), prolonged mechanical ventilation 1.38 (95% CI 1.18–1.62), vasopressor requirement 1.34 (95% CI 1.16–1.55), RRT 1.49 (95% CI 1.10–2.01), and 90-day readmission 1.27 (95% CI 1.07–1.51). Cumulative incidence at 30 and 90 days is reported in Table 2 as fixed-horizon matched risks.

### 3.6. Dose–Response Analysis by Cardiac Comorbidity Burden

A significant dose–response relationship was observed between the number of pre-transplant cardiac comorbidity domains and adverse outcomes. The 30-day mortality, 90-day mortality, 30-day MACE, 7-day AKI, and 7-day prolonged mechanical ventilation all increased in a stepwise fashion across comorbidity burden categories (0, 1, 2, 3). The Cochran–Armitage test for trend was significant for all outcomes examined (Table 5).

### 3.7. Individual Cardiac Comorbidity Analysis

Separate propensity-matched analyses were performed for each cardiac comorbidity (CAD, HF, AF) vs. no cardiac comorbidity. In each of the three matches, all 16 covariates were balanced after matching (maximum post-match SMD 0.061 in the CAD match, 0.072 in the HF match, and 0.068 in the AF match; all pre-match maximum SMDs exceeded 0.30, driven by age, hypertension, and type 2 diabetes). Matched sample sizes were 578 pairs for CAD, 336 pairs for HF, and 379 pairs for AF.

In these separate matched analyses, CAD was most strongly associated with mortality (30-day RR 1.94), HF with AKI and RRT, and AF with MACE (30-day RR 2.31). All three comorbidities were associated with prolonged mechanical ventilation and vasopressor requirement in separate matched analyses. Because 264 patients had two comorbidity domains and 74 had all three, individuals could contribute to more than one comparison, and these estimates are not mutually adjusted; they should not be interpreted as independent effects of each condition. A mutually adjusted model was not fitted because the overlapping comorbidity structure and modest stratum sizes would not support stable simultaneous estimation (Table 6).

### 3.8. MASH vs. Non-MASH Subgroup Analysis

To explore whether cardiac comorbidity–outcome associations differed by underlying liver disease etiology, outcomes were examined within MASH-cirrhosis (*n* = 218 cardiac, *n* = 218 no cardiac, after subgroup-specific PSM) and non-MASH (*n* = 756 cardiac, *n* = 756 no cardiac) subgroups. MASH-cirrhosis recipients with cardiac comorbidities had numerically higher event rates than did non-MASH recipients with cardiac comorbidities across most outcomes. However, interaction *p*-values were non-significant for most outcomes, indicating no statistically detectable heterogeneity between MASH and non-MASH subgroups. These analyses were exploratory and were not powered for definitive subgroup-specific conclusions (Table 7). 

However, no interaction reached statistical significance, and all interaction estimates were imprecise, with confidence intervals that included the null and extended well into both directions (ratio of relative risks 1.16 to 1.33; all upper bounds ≥ 2.1). These data therefore do not provide statistical evidence that the effect of cardiac comorbidity differs by MASH status. The subgroup analyses were exploratory, were not powered for subgroup-specific inference, and were not adjusted for multiplicity.

### 3.9. MELD 3.0 Sensitivity Analysis

To evaluate whether findings were robust to the use of a contemporary liver disease severity metric, PSM was repeated after substituting MELD 3.0 for MELD-Na in the propensity score model. The direction and magnitude of associations remained broadly consistent with the primary analysis across all outcomes (Table 8). Balance was again adequate in this match (maximum post-match SMD 0.048).

### 3.10. Strict MASH Definition Sensitivity Analysis

To address potential misclassification arising from the metabolic-risk-factor-plus-cirrhosis criterion, the MASH subgroup analysis was repeated using only the definite MASH definition (K75.81 with a cirrhosis code; *n* = 187, of whom 94 had cardiac comorbidity). After subgroup-specific matching (91 pairs), point estimates were directionally consistent with the primary MASH analysis but substantially less precise: 30-day mortality RR 2.11 (95% CI 0.83–5.37), 30-day restricted hard MACE RR 1.82 (95% CI 0.86–3.85), and 7-day AKI RR 1.49 (95% CI 0.99–2.24). No interaction with non-MASH recipients was statistically significant. Reclassifying probable-MASH patients as other/cryptogenic did not materially change any primary or secondary outcome estimate in the full matched cohort (all relative risks within 0.05 of the primary analysis).

## 4. Discussion

In this large propensity-matched multicenter cohort of 1948 liver transplant recipients, pre-transplant cardiac comorbidity burden was associated with worse early post-transplant outcomes across multiple domains, including mortality, MACE, AKI, prolonged mechanical ventilation, vasopressor requirement, RRT, prolonged hospitalization, and 90-day readmission. The association with cardiac events persisted when the composite was restricted to hard endpoints that cannot overlap with the exposure definitions, though it was attenuated, indicating that part of the broad composite signal reflects postoperative HF and arrhythmia coding in patients whose baseline cardiac disease defined the exposure. MASH-cirrhosis recipients with cardiac comorbidities had numerically higher event rates than did non-MASH recipients across most outcomes, although all interaction estimates were compatible with no difference.

The mortality findings are consistent with prior literature demonstrating the adverse prognostic impact of CAD on post-transplant survival. A single-center study of 693 LT recipients reported that pre-existing CAD was associated with significantly higher all-cause mortality (26.7% vs. 9.6%; *p* < 0.01), with cardiovascular events accounting for 52.5% of deaths in the CAD group [9]. A meta-analysis of 15,880 LT recipients found that CAD was associated with increased post-transplant MACE and mortality, with any degree of coronary disease conferring elevated risk [10,11]. The present study extends these findings by demonstrating that the cumulative burden of cardiac comorbidities—not just CAD alone—confers incrementally higher risk, although the highest-burden stratum was small (*n* = 74), and its estimates are imprecise. 

In separate matched analyses, differential patterns emerged across comorbidity types. CAD was most strongly associated with mortality (30-day RR 1.94), consistent with its established role as a predictor of post-transplant death and its inclusion in the LTCI [7]. AF carried the highest relative risk for MACE (30-day RR 2.31), directionally consistent with meta-analytic data reporting increased mortality (OR 2.34; 95% CI 1.10–5.00) and cardiovascular complications (OR 5.15; 95% CI 2.67–9.92) among recipients with pre-existing AF [13]. HF was most strongly associated with AKI and RRT, which may reflect cardiorenal interactions involving reduced cardiac output and renal hypoperfusion in the perioperative setting, although this study contains no hemodynamic nor echocardiographic data with which to test that mechanism [5,6]. Because these three matched analyses share patients and are not mutually adjusted, apparent differences between comorbidities should be regarded as descriptive rather than as evidence that one condition independently confers greater risk than another [14].

The MASH subgroup analysis was exploratory and did not demonstrate statistically detectable heterogeneity. Event rates were numerically higher among MASH-cirrhosis recipients with cardiac comorbidity than among non-MASH recipients, but every interaction confidence interval included the null, the strict-definition sensitivity analysis was underpowered, and the etiology was coding-derived rather than clinically adjudicated. These observations constitute an unconfirmed descriptive hypothesis and do not support etiology-specific changes to pre-transplant cardiac evaluation. Adequately powered MASH-specific cohorts with adjudicated etiology are required [12].

The dose–response pattern between cardiac comorbidity burden and adverse outcomes is a novel descriptive observation of this study. The stepwise increase in event rates from 0 to 3 comorbidity domains is consistent with a cumulative risk model rather than with a threshold effect, but it was derived from unmatched, unadjusted comparisons in which the highest-burden stratum contained few events. Current composite indices such as the LTCI incorporate CAD as a binary variable and do not account for concurrent HF or AF; whether a cardiac comorbidity burden term would improve discrimination beyond that of existing indices was not tested here and remains an open question for prospective model development.

The association between cardiac comorbidities and AKI was supported by the continuous renal endpoints, with higher peak creatinine and a larger rise from baseline to peak in the cardiac cohort. Because coded AKI is likely to capture predominantly clinically recognized moderate-to-severe events, these continuous measures should be regarded as the more reliable renal signal in this dataset. Potential contributors include perioperative hemodynamic instability, increased vasopressor exposure, and calcineurin inhibitor nephrotoxicity, none of which could be assessed directly in this analysis [15].

These findings have several clinical implications. First, they support comprehensive pre-transplant cardiac risk stratification that accounts for the cumulative burden of cardiac comorbidities rather than evaluating each condition in isolation [16]. The 2025 AASLD/AST Practice Guideline recommends a stepwise approach to CAD assessment but does not provide specific guidance on the cumulative impact of multiple cardiac comorbidities [12]. Second, the differential risk profiles of CAD, HF, and AF suggest that perioperative management strategies may need to be tailored to the specific cardiac comorbidity profile [17]. Third, the numerically higher event rates in MASH-cirrhosis recipients with cardiac comorbidities support intensified cardiac evaluation in this growing population [18]. Fourth, the association of cardiac comorbidities with AKI, prolonged mechanical ventilation, and hemodynamic instability identifies specific early outcomes that may be amenable to targeted perioperative optimization, including enhanced hemodynamic monitoring, nephroprotective protocols, and respiratory support planning [19,20,21]. Fifth, the dose–response relationship suggests that a cardiac comorbidity burden score could enhance existing risk stratification tools such as the LTCI [4]. However, these implications remain hypothesis-generating, and the present observational analysis cannot establish that intensified cardiac assessment, risk stratification, or perioperative optimization improves post-transplant outcomes.

These implications are strengthened by several features of the present study. The TriNetX platform provided a large, geographically diverse, multicenter cohort that is broadly representative of US transplant centers. The sample size (974 matched pairs) enabled assessment of multiple secondary outcomes with adequate statistical power. The comprehensive propensity matching using 16 covariates provided robust confounding adjustment. The dose–response analysis, individual cardiac comorbidity analysis, and MASH vs. non-MASH subgroup analysis provide complementary perspectives on the cardiac comorbidity–outcome relationship. The MELD 3.0 sensitivity analysis supports the robustness of the findings. 

Despite these strengths, several limitations warrant consideration. First, cardiac comorbidities were identified using ICD-10-CM administrative codes rather than echocardiographic, angiographic, or functional assessment, so disease severity (ejection fraction, number of diseased vessels, AF burden) could not be characterized, and subclinical disease is likely underascertained. Second, propensity score matching balanced only measured covariates and could not balance several major determinants of early post-transplant outcome that are unavailable in this database, including donor age and donor type (DCD vs. DBD); graft quality, cold and warm ischemia times; intraoperative blood loss and transfusion requirement; waitlist urgency and status at transplant; center volume and center-specific perioperative practice; pre-transplant cardiac testing results and disease severity; functional status and frailty; and pre- and post-transplant medication exposure, including beta-blockers, antiplatelet agents, statins, anticoagulants, and immunosuppressive regimen. Confounding adjustment in this study is therefore partial, and residual confounding by these factors is likely rather than merely possible. Third, a post-index diagnosis code alone cannot reliably distinguish recurrent administrative coding of a chronic pre-existing condition from a genuinely incident event or true clinical decompensation. Although postoperative HF and arrhythmia were counted only when incident or clinically active, the broad MACE composite remains susceptible to inflation because baseline HF and AF also define the exposure. The restricted hard-endpoint composite should therefore be regarded as the primary cardiac outcome and the broad composite as supportive. Fourth, several outcomes were identified using structured EHR and administrative data rather than manual chart adjudication. AKI was defined using ICD-10-CM codes rather than KDIGO criteria, which likely underestimates mild AKI. Because serial creatinine timestamps are incompletely available across the network, the direction and magnitude of this bias cannot be verified, and the assumption of nondifferential misclassification is plausible but not demonstrable. Vasopressor requirement and mechanical ventilation duration may be imprecisely captured. Fifth, the federated EHR design creates the possibility of incomplete longitudinal follow-up, coding variation among institutions, and outcome misclassification; events occurring outside participating organizations may not be captured, which particularly affects post-discharge outcomes such as readmission. Deaths outside the network may also be missed, which would bias mortality estimates toward the null if underascertainment is nondifferential. Sixth, several outcome variables—ICU length of stay, peak creatinine, and ventilator duration—were analyzed only among patients in whom they were recorded, which depends on survival and on receipt of the intervention and may introduce selection bias. Seventh, no formal multiplicity control was applied to the primary analysis. Multiple outcomes, time points, individual exposures, dose–response tests, and subgroup analyses were examined, and although FDR adjustment did not materially change conclusions for the secondary outcome family, the exploratory analyses should be interpreted accordingly. Eighth, etiology was coding-derived rather than clinically adjudicated. The hierarchical algorithm excluded patients with viral or alcohol-related codes from MASH classification, but cryptogenic cirrhosis and mixed-etiology disease may still be misclassified as MASH, particularly among patients meeting only the probable definition. Ninth, exclusion of patients missing three or more matching variables (*n* = 143) may have induced selection bias, as missingness may relate to referral pattern, acuity, and completeness of data contribution by individual healthcare organizations. Tenth, the retrospective nature of the TriNetX data source imposes limitations inherent to large administrative and EHR-based databases. Data are collected primarily for routine clinical care, billing, and operational purposes rather than according to a prospectively standardized research protocol. Consequently, the accuracy and completeness of diagnoses, procedures, medication exposures, and outcomes depend on local documentation and coding practices, and clinically relevant information may be absent, inconsistently recorded, or misclassified. Variation in coding intensity across participating healthcare organizations and the lack of source-document or manual chart adjudication may introduce additional measurement error. These limitations cannot be fully addressed through propensity score matching or other statistical adjustment. Finally, given the observational design and administrative data source, all findings should be interpreted as associative rather than causal.

## 5. Conclusions

Pre-transplant cardiac comorbidity burden was associated with worse early outcomes after liver transplantation, including mortality, cardiac events, renal complications, prolonged mechanical ventilation, hemodynamic instability, longer hospitalization, and readmission. The association with cardiac events persisted, although attenuated, when restricted to hard endpoints. Apparent differences among CAD, HF, and AF and the stepwise increase across comorbidity-burden categories were descriptive, while exploratory MASH analyses did not demonstrate statistically significant heterogeneity. These findings are associative and hypothesis-generating. Prospective studies incorporating detailed cardiac, donor, operative, frailty, medication, and center-level data are needed to validate these associations and determine whether improved risk prediction or targeted perioperative strategies can modify post-transplant outcomes.

## Figures and Tables

**Table 1:** Baseline demographic and clinical characteristics before and after propensity score matching.

Characteristic	Before PSM Cardiac (N = 986)	Before PSM No Cardiac (N = 4138)	Before PSM *p* Value	Before PSM SMD	After PSM Cardiac (N = 974)	After PSM No Cardiac (N = 974)	After PSM *p* Value	After PSM SMD
Age, years	62.4 ± 9.8	55.7 ± 11.1	<0.001	0.640	62.2 ± 9.7	61.9 ± 10.0	0.502	0.030
Female	298 (30.2%)	1407 (34.0%)	0.024	0.081	296 (30.4%)	302 (31.0%)	0.768	0.013
White	694 (70.4%)	2731 (66.0%)	0.009	0.094	685 (70.3%)	679 (69.7%)	0.767	0.013
Black	128 (13.0%)	601 (14.5%)	0.213	0.045	127 (13.0%)	131 (13.4%)	0.789	0.012
Hispanic	108 (11.0%)	558 (13.5%)	0.034	0.077	107 (11.0%)	112 (11.5%)	0.720	0.016
Type 2 diabetes	468 (47.5%)	1324 (32.0%)	<0.001	0.316	461 (47.3%)	453 (46.5%)	0.716	0.016
Hypertension	672 (68.2%)	2068 (50.0%)	<0.001	0.370	663 (68.1%)	656 (67.4%)	0.734	0.015
CKD	347 (35.2%)	1035 (25.0%)	<0.001	0.222	341 (35.0%)	334 (34.3%)	0.739	0.015
Hepatic encephalopathy	462 (46.9%)	1779 (43.0%)	0.028	0.078	456 (46.8%)	449 (46.1%)	0.750	0.014
Ascites	558 (56.6%)	2316 (56.0%)	0.723	0.013	551 (56.6%)	546 (56.1%)	0.819	0.010
HCC	201 (20.4%)	879 (21.2%)	0.553	0.021	199 (20.4%)	203 (20.8%)	0.823	0.010
Obesity	384 (38.9%)	1159 (28.0%)	<0.001	0.232	378 (38.8%)	371 (38.1%)	0.744	0.015
Hyperlipidemia	347 (35.2%)	910 (22.0%)	<0.001	0.292	341 (35.0%)	335 (34.4%)	0.775	0.013
Pre-transplant dialysis	108 (11.0%)	311 (7.5%)	<0.001	0.119	105 (10.8%)	101 (10.4%)	0.768	0.013
MELD-Na	23.9 ± 7.1	22.4 ± 6.7	<0.001	0.217	23.8 ± 7.0	23.6 ± 6.9	0.525	0.029
Creatinine, mg/dL	1.48 ± 0.78	1.27 ± 0.66	<0.001	0.291	1.47 ± 0.77	1.45 ± 0.74	0.559	0.026
Bilirubin, mg/dL	6.8 ± 5.9	6.6 ± 5.8	0.337	0.034	6.7 ± 5.8	6.8 ± 5.7	0.701	0.017
INR	1.82 ± 0.54	1.79 ± 0.53	0.116	0.056	1.81 ± 0.54	1.80 ± 0.53	0.680	0.019
Sodium, mmol/L	132.1 ± 5.4	132.4 ± 5.3	0.116	0.056	132.1 ± 5.3	132.3 ± 5.2	0.401	0.038
Albumin, g/dL	2.74 ± 0.48	2.82 ± 0.46	<0.001	0.170	2.75 ± 0.48	2.76 ± 0.47	0.642	0.021

Abbreviations: CKD = chronic kidney disease; HCC = hepatocellular carcinoma; INR = international normalized ratio; MELD-Na = Model for End-Stage Liver Disease-Sodium; PSM = propensity score matching; SMD = standardized mean difference. Continuous variables are presented as mean ± standard deviation and categorical variables as *n* (%). *p* values were calculated using Chi-square tests for categorical variables and two-sample *t*-tests for continuous variables. Standardized mean differences < 0.10 after matching were considered indicative of adequate covariate balance. Race and ethnicity were extracted from structured EHR fields and were not mutually exclusive. Patients with unknown or not-recorded race (*n* = 56 [5.7%], cardiac; *n* = 249 [6.0%], no cardiac) and those in categories other than White, Black, or Hispanic (*n* = 62 [6.3%], cardiac; *n* = 268 [6.5%], no cardiac) are not displayed; percentages therefore do not sum to 100%. Race indicators were entered into the propensity model with “unknown” treated as a separate category. Bilirubin, INR, creatinine, and sodium levels were not included in the propensity score model and are shown as post-match balance diagnostics only. Pre- and post-match SMDs are shown for every variable in the columns provided.

**Table 2:** (**A**) Relative risks and risk differences for clinical outcomes after propensity score matching. (**B**) Components of the restricted and broad MACE composites after propensity score matching.

(**A**)						
**Outcome**	**Time Point**	**Cardiac (*n*/N)**	**No Cardiac (*n*/N)**	**RD (95% CI)**	**RR (95% CI)**	***p* Value**
All-cause mortality	30 Days	62/974	34/974	0.029 (0.010–0.048)	1.82 (1.28–2.59)	<0.003
All-cause mortality	90 Days	98/974	61/974	0.038 (0.014, 0.062)	1.61 (1.24–2.09)	<0.002
MACE	30 Days	134/974	68/974	0.068 (0.039, 0.097)	1.97 (1.51–2.57)	<0.001
MACE	90 Days	178/974	102/974	0.078 (0.046, 0.110)	1.75 (1.40–2.18)	<0.001
Acute kidney injury	7 Days	284/974	198/974	0.088 (0.047, 0.129)	1.43 (1.23–1.67)	<0.001
Acute kidney injury	30 Days	351/974	268/974	0.085 (0.041, 0.129)	1.31 (1.16–1.48)	<0.001
Acute kidney injury	90 Days	392/974	318/974	0.076 (0.031, 0.121)	1.23 (1.11–1.37)	<0.001
Prolonged mechanical ventilation	7 Days	237/974	162/974	0.077 (0.038, 0.116)	1.46 (1.23–1.73)	<0.001
Prolonged mechanical ventilation	30 Days	261/974	189/974	0.074 (0.033, 0.115)	1.38 (1.18–1.62)	<0.001
Vasopressor requirement/hemodynamic instability	7 Days	296/974	208/974	0.090 (0.049, 0.131)	1.42 (1.23–1.65)	<0.001
Vasopressor requirement/hemodynamic instability	30 Days	318/974	237/974	0.083 (0.040, 0.126)	1.34 (1.17–1.54)	<0.001
Renal replacement therapy	7 Days	68/974	39/974	0.030 (0.010, 0.050)	1.74 (1.18–2.57)	0.004
Renal replacement therapy	30 Days	94/974	58/974	0.037 (0.013, 0.061)	1.62 (1.17–2.24)	0.003
90-day readmission	90 Days	214/974	162/974	0.053 (0.016, 0.090)	1.32 (1.11–1.57)	0.002
(**B**)						
**Component**	**Time Point**	**Cardiac (N = 974)**	**No Cardiac (N = 974)**	**RR (95% CI)**		
All-cause death	30 days	62 (6.4%)	34 (3.5%)	1.82 (1.21–2.74)		
Myocardial infarction (I21.x)	30 days	22 (2.3%)	11 (1.1%)	2.00 (0.97–4.11)		
Cardiac arrest (I46.x)	30 days	26 (2.7%)	14 (1.4%)	1.86 (0.98–3.53)		
Ischemic stroke (I63.x)	30 days	14 (1.4%)	9 (0.9%)	1.56 (0.68–3.58)		
Restricted “hard” MACE	30 days	84 (8.6%)	51 (5.2%)	1.65 (1.18–2.30)		
Restricted “hard” MACE	90 days	121 (12.4%)	78 (8.0%)	1.55 (1.19–2.03)		
Incident postoperative HF	90 days	47 (4.8%)	24 (2.5%)	1.96 (1.21–3.17)		
Incident or clinically active postoperative arrhythmia	90 days	58 (6.0%)	31 (3.2%)	1.87 (1.22–2.86)		
Broad MACE composite	90 days	178 (18.3%)	102 (10.5%)	1.75 (1.40–2.18)		

Abbreviations: CI = confidence interval; HF = heart failure; MACE = major adverse cardiac events; RD = risk difference; RR = relative risk. Components are not mutually exclusive; patients experiencing more than one component are counted once in each applicable component row and once in the composite. Postoperative HF and arrhythmia were counted only when incident (no corresponding code during the 12-month baseline window) or clinically active (inpatient encounter with first-listed cardiac diagnosis or cardiac-specific intervention).

**Table 3:** Length of stay and continuous outcomes after propensity score matching, with analysis denominators and distributional summaries.

Outcome	Analysis Population (Cardiac/No Cardiac)	Cardiac, Mean ± SD	No Cardiac, Mean ± SD	Cardiac, Median (IQR)	No Cardiac, Median (IQR)	Median Difference (95% CI)	*p* Value
Hospital length of stay, days	974/974	19.4 ± 10.8	15.2 ± 8.6	17 (12–24)	13 (9–19)	4.0 (3.0–5.0)	<0.001
ICU length of stay, days	921/903	7.8 ± 5.2	5.7 ± 4.0	6 (4–10)	5 (3–7)	1.5 (1.0–2.0)	<0.001
Peak creatinine during admission, mg/dL	948/941	2.24 ± 1.14	1.82 ± 0.93	1.94 (1.42–2.76)	1.60 (1.18–2.21)	0.34 (0.26–0.42)	<0.001
Change in creatinine, baseline to peak, mg/dL	942/936	0.76 ± 0.81	0.37 ± 0.58	0.58 (0.22–1.10)	0.28 (0.05–0.62)	0.30 (0.24–0.36)	<0.001
Ventilator days among ventilated patients	261/189	6.4 ± 4.7	4.2 ± 3.4	5 (3–8)	3 (2–5)	2.0 (1.0–2.0)	<0.001

Abbreviations: CI = confidence interval; ICU = intensive care unit. Continuous variables are presented as mean ± standard deviation. Denominators differ by outcome. ICU length of stay is restricted to patients with a recorded ICU encounter; peak and delta creatinine to patients with at least two recorded inpatient creatinine values; ventilator days to mechanically ventilated patients only. These conditional analyses are susceptible to selection by survival and by receipt of the intervention and should not be interpreted as representative of the full matched cohort. Median differences were estimated by quantile regression accounting for matched pairs.

**Table 4:** Cox proportional hazards models for outcomes associated with pre-transplant cardiac comorbidity in the propensity-matched cohort.

Outcome	HR	Coefficient	SE	z	*p* Value	95% CI
**All-cause mortality**	1.64	0.495	0.132	3.75	<0.001	1.27–2.12
**MACE**	1.78	0.576	0.108	5.33	<0.001	1.44–2.20
**Acute kidney injury**	1.36	0.307	0.062	4.95	<0.001	1.21–1.53
**Prolonged mechanical ventilation**	1.44	0.365	0.078	4.68	<0.001	1.24–1.68
**Vasopressor requirement/hemodynamic instability**	1.39	0.329	0.071	4.63	<0.001	1.21–1.60
**Renal replacement therapy**	1.58	0.457	0.148	3.09	0.002	1.18–2.12
**90-day readmission**	1.34	0.293	0.084	3.49	<0.001	1.14–1.58

Abbreviations: CI = confidence interval; HR = hazard ratio; MACE = major adverse cardiac events; SE = standard error. Hazard ratios were estimated with robust (sandwich) standard errors clustered on a matched pair; corresponding Fine–Gray subdistribution hazard ratios for nonfatal outcomes are reported in Section 3.5.

**Table 5:** Dose–response analysis: outcomes by number of pre-transplant cardiac comorbidity domains (0, 1, 2, or 3) in the unmatched cohort.

Outcome	0 Comorbidities (N = 4138)	1 Comorbidity (N = 648)	2 Comorbidities (N = 264)	3 Comorbidities (N = 74)	*p* for Trend
**30-day mortality, *n* (%)**	128 (3.1%)	34 (5.2%)	21 (8.0%)	9 (12.2%)	<0.001
**90-day mortality, *n* (%)**	228 (5.5%)	56 (8.6%)	32 (12.1%)	13 (17.6%)	<0.001
**30-day MACE, *n* (%)**	254 (6.1%)	72 (11.1%)	46 (17.4%)	19 (25.7%)	<0.001
**7-day AKI, *n* (%)**	786 (19.0%)	162 (25.0%)	88 (33.3%)	31 (41.9%)	<0.001
**7-day prolonged MV, *n* (%)**	621 (15.0%)	132 (20.4%)	74 (28.0%)	26 (35.1%)	<0.001
**7-day vasopressor requirement, *n* (%)**	828 (20.0%)	168 (25.9%)	92 (34.8%)	30 (40.5%)	<0.001
**30-day RRT, *n* (%)**	228 (5.5%)	52 (8.0%)	30 (11.4%)	12 (16.2%)	<0.001
**90-day readmission, *n* (%)**	621 (15.0%)	118 (18.2%)	64 (24.2%)	22 (29.7%)	<0.001

Abbreviations: AKI = acute kidney injury; MACE = major adverse cardiac events; MV = mechanical ventilation; RRT = renal replacement therapy. Cochran–Armitage test for trend was used to assess dose–response across comorbidity burden categories. Estimates are unadjusted. The three-comorbidity stratum (*n* = 74) yielded 9–31 events per outcome and is subject to substantial sampling variability; corresponding 95% confidence intervals for 30-day mortality in this stratum span 5.7% to 21.8%. These trend analyses are exploratory and were not adjusted for multiplicity.

**Table 6:** Individual cardiac comorbidity analyses: matched sample sizes, relative risks, risk differences, and exact *p* values for each outcome in three separate propensity-matched comparisons vs. no cardiac comorbidity.

Outcome	Time Point	CAD vs. None (578 Pairs) RR (95% CI); RD; *p*	HF vs. None (336 Pairs) RR (95% CI); RD; *p*	AF vs. None (379 Pairs) RR (95% CI); RD; *p*
All-cause mortality	30 days	1.94 (1.24–3.04); +3.3%; *p* = 0.0038	1.78 (1.08–2.93); +2.9%; *p* = 0.0231	1.67 (1.02–2.74); +2.6%; *p* = 0.0417
All-cause mortality	90 days	1.72 (1.22–2.42); +4.4%; *p* = 0.0019	1.58 (1.08–2.31); +3.9%; *p* = 0.0182	1.49 (1.01–2.20); +3.2%; *p* = 0.0442
Restricted hard MACE	30 days	1.58 (1.09–2.29); +3.5%; *p* = 0.0153	1.71 (1.13–2.59); +4.2%; *p* = 0.0110	1.86 (1.24–2.79); +4.8%; *p* = 0.0026
Broad MACE	30 days	1.84 (1.32–2.56); +5.7%; *p* = 0.0003	2.12 (1.48–3.04); +7.4%; *p* < 0.0001	2.31 (1.62–3.30); +8.3%; *p* < 0.0001
Broad MACE	90 days	1.68 (1.27–2.22); +7.1%; *p* = 0.0002	1.89 (1.39–2.57); +8.9%; *p* < 0.0001	2.04 (1.50–2.78); +9.8%; *p* < 0.0001
AKI	7 days	1.38 (1.14–1.67); +7.2%; *p* = 0.0009	1.52 (1.24–1.86); +9.8%; *p* < 0.0001	1.41 (1.14–1.74); +7.9%; *p* = 0.0014
Prolonged MV	7 days	1.42 (1.14–1.77); +6.3%; *p* = 0.0018	1.48 (1.17–1.87); +7.1%; *p* = 0.0010	1.51 (1.19–1.92); +7.5%; *p* = 0.0007
Vasopressor requirement	7 days	1.38 (1.14–1.67); +7.6%; *p* = 0.0010	1.46 (1.19–1.79); +9.0%; *p* = 0.0003	1.44 (1.16–1.78); +8.7%; *p* = 0.0009
RRT	30 days	1.54 (1.02–2.32); +2.9%; *p* = 0.0392	1.78 (1.16–2.73); +4.1%; *p* = 0.0084	1.62 (1.04–2.52); +3.4%; *p* = 0.0331
90-day readmission	90 days	1.28 (1.03–1.59); +4.0%; *p* = 0.0261	1.38 (1.09–1.75); +5.5%; *p* = 0.0075	1.34 (1.06–1.70); +4.9%; *p* = 0.0141

Abbreviations: AF = atrial fibrillation; AKI = acute kidney injury; CAD = coronary artery disease; CI = confidence interval; HF = heart failure; MACE = major adverse cardiac events; MV = mechanical ventilation; RD = risk difference; RR = relative risk; RRT = renal replacement therapy. Each column represents a separate 1:1 propensity score match against the no-cardiac-comorbidity cohort using the same 16 covariates. Maximum post-match standardized mean difference was 0.061 (CAD), 0.072 (HF), and 0.068 (AF). Patients with more than one cardiac comorbidity contribute to more than one column; estimates are not mutually adjusted. *p* values are unadjusted for multiplicity; these analyses are exploratory.

**Table 7:** MASH vs. non-MASH subgroup analysis: selected outcomes in recipients with cardiac comorbidities after subgroup-specific propensity score matching, with interaction estimates and 95% confidence intervals.

Outcome	Time Point	MASH RR (95% CI)	Non-MASH RR (95% CI)	Ratio of RRs (95% CI)	Interaction *p*
**All-cause mortality**	30 days	2.25 (1.00–5.06)	1.69 (1.06–2.70)	1.33 (0.55–3.23)	0.482
**All-cause mortality**	90 days	2.00 (1.08–3.70)	1.49 (1.05–2.12)	1.34 (0.71–2.55)	0.361
Broad **MACE**	30 days	2.38 (1.37–4.12)	1.85 (1.34–2.55)	1.29 (0.70–2.36)	0.418
Restricted hard MACE	30 days	1.94 (1.02–3.69)	1.58 (1.08–2.31)	1.23 (0.61–2.48)	0.561
**AKI**	7 days	1.57 (1.13–2.17)	1.39 (1.17–1.66)	1.13 (0.79–1.62)	0.524
**Prolonged MV**	7 days	1.63 (1.14–2.34)	1.41 (1.15–1.73)	1.16 (0.78–1.71)	0.487
**RRT**	30 days	2.00 (1.08–3.70)	1.50 (1.04–2.16)	1.33 (0.68–2.62)	0.378
**90-day readmission**	90 days	1.47 (1.02–2.13)	1.27 (1.03–1.57)	1.16 (0.77–1.75)	0.492

Abbreviations: AKI = acute kidney injury; CI = confidence interval; MACE = major adverse cardiac events; MASH = metabolic dysfunction-associated steatohepatitis; MV = mechanical ventilation; RR = relative risk; RRT = renal replacement therapy. Interaction was assessed on the multiplicative scale; the ratio of relative risks compares the cardiac comorbidity effect in MASH vs. non-MASH recipients, with the null value of 1.00 contained within every confidence interval. Event counts by cell are as previously reported. These analyses are exploratory and unadjusted for multiplicity.

**Table 8:** Sensitivity analysis using MELD 3.0 instead of MELD-Na in the propensity score matching model.

Outcome	Time Point	Cardiac *n*/N	No Cardiac *n*/N	RD, %	RR (95% CI)	*p* Value
All-cause mortality	30 Days	60/962	33/962	2.8	1.82 (1.21–2.73)	0.003
All-cause mortality	90 Days	94/962	59/962	3.6	1.59 (1.17–2.17)	0.003
MACE	30 Days	130/962	66/962	6.7	1.97 (1.49–2.60)	0.001
MACE	90 Days	174/962	100/962	7.7	1.74 (1.39–2.18)	0.001
AKI	7 Days	278/962	196/962	8.5	1.42 (1.22–1.65)	0.001
AKI	30 Days	344/962	264/962	8.3	1.30 (1.15–1.48)	0.001
Prolonged MV	7 Days	232/962	160/962	7.5	1.45 (1.22–1.72)	0.001
Vasopressor requirement	7 Days	290/962	206/962	8.7	1.41 (1.21–1.64)	0.001
RRT	7 Days	66/962	38/962	2.9	1.74 (1.17–2.58)	0.005
RRT	30 Days	91/962	57/962	3.5	1.60 (1.15–2.22)	0.005
90-day readmission	90 Days	210/962	160/962	5.2	1.31 (1.10–1.57)	0.003

Abbreviations: AKI = acute kidney injury; CI = confidence interval; MACE = major adverse cardiac events; MELD = Model for End-Stage Liver Disease; MV = mechanical ventilation; RD = risk difference; RR = relative risk; RRT = renal replacement therapy.

## Data Availability

The data that support the findings of this study are available from TriNetX, but restrictions apply to the availability of these data, which were used under license for the current study and are therefore not publicly available. Data may be available from the authors upon reasonable request and with permission of TriNetX.

## Abbreviations

The following abbreviations are used in this manuscript:

AASLD	American Association for the Study of Liver Diseases
AF	Atrial fibrillation
AKI	Acute kidney injury
AST	American Society of Transplantation
CAD	Coronary artery disease
CI	Confidence interval
CKD	Chronic kidney disease
CPT	Current Procedural Terminology
DBD	Donation after brain death
DCD	Donation after circulatory death
EHR	Electronic health record
HCC	Hepatocellular carcinoma
HF	Heart failure
HIPAA	Health Insurance Portability and Accountability Act
HR	Hazard ratio
ICD-10-CM	International Classification of Diseases, Tenth Revision, Clinical Modification
ICD-10-PCS	International Classification of Diseases, Tenth Revision, Procedure Coding System
ICU	Intensive care unit
INR	International normalized ratio
KDIGO	Kidney Disease: Improving Global Outcomes
LOS	Length of stay
LT	Liver transplantation
LTCI	Liver Transplant Comorbidity Index
MACE	Major adverse cardiac events
MASH	Metabolic dysfunction-associated steatohepatitis
MASLD	Metabolic dysfunction-associated steatotic liver disease
MELD 3.0	Model for End-Stage Liver Disease 3.0
MELD-Na	Model for End-Stage Liver Disease-Sodium
MV	Mechanical ventilation
OPTN	Organ Procurement and Transplantation Network
PSM	Propensity score matching
RD	Risk difference
RR	Relative risk
RRT	Renal replacement therapy
SMD	Standardized mean difference
STROBE	Strengthening the Reporting of Observational Studies in Epidemiology
